# Loneliness during the COVID-19 Pandemic: A Comparison between Older and Younger People

**DOI:** 10.3390/ijerph18157871

**Published:** 2021-07-25

**Authors:** Mostafa Saidur Rahim Khan, Yoshihiko Kadoya

**Affiliations:** School of Economics, Hiroshima University, 1-2-1 Kagamiyama Higashihiroshima, Hiroshima 739-8525, Japan; ykadoya@hiroshima-u.ac.jp

**Keywords:** loneliness, COVID-19 pandemic, social isolation, older and younger people, socio-demographic and psychological factors, comparative analysis, logit regression, Japan

## Abstract

The precautionary measures and uncertainties surrounding the COVID-19 pandemic have serious psychological impacts on peoples’ mental health. We used longitudinal data from Hiroshima University to investigate loneliness before and during the pandemic among older and younger people in Japan. We provide evidence that loneliness among both older and younger people increased considerably during the pandemic. Although loneliness among younger people is more pervasive, the magnitude of increase in loneliness during the pandemic is higher among older people. Our logit regression analysis shows that age, subjective health status, and feelings of depression are strongly associated with loneliness before and during the pandemic. Moreover, household income and financial satisfaction are associated with loneliness among older people during the pandemic while gender, marital status, living condition, and depression are associated with loneliness among younger people during the pandemic. The evidence of increasing loneliness during the pandemic is concerning for a traditionally well-connected and culturally collectivist society such as Japan. As loneliness has a proven connection with both physical and mental health, we suggest immediate policy interventions to provide mental health support for lonely people so they feel more cared for, secure, and socially connected.

## 1. Introduction

The COVID-19 pandemic had a profound impact on many aspects of peoples’ psychological conditions, including loneliness. Several studies report that loneliness among both younger and older people has considerably increased during the pandemic due to maintaining virus mitigating measures such as social distancing, self-isolation, shelter in place, work from home, etc. [1,2,3,4,5,6,7]. The uncertainties and mitigating measures surrounding the pandemic have changed peoples’ normal lifestyle and social relationships in such a way that their psychological conditions and loneliness have become vulnerable. The concern about loneliness among older people is particularly worrying due to their living status, need for long-term care, and vulnerable physical and mental health conditions [8,9,10].

Loneliness is a psycho-sociological state of mind in which a person feels a lack of companionship or social connectedness quantitatively, qualitatively, or both. Social connectedness is a fundamental requirement that provides mental soundness. The literature defines loneliness as the unpleasant subjective experience that occurs when a person’s desire for social relations is deficient, either quantitatively or qualitatively [11,12,13]. Loneliness is not similar to living alone or social isolation, although these factors could contribute to loneliness. As a subjective feeling, people may experience loneliness even when they are physically accompanied and socially connected. Cacioppo and Patrick [14] argue that the mere presence of others does not make people feel less lonely; rather, they need the presence of someone whom they trust and can share common goals, plan for future, and work together to survive and prosper. In this study, we perceive loneliness as peoples’ subjective feeling of being isolated, left out, and lack of companionship. Loneliness appears across various demographic and socio-economic groups [15,16] and is intensifying due to social issues such as lower rates of marriage, fewer children in families, and shrinking household sizes [15,16,17]. The recent COVID-19 pandemic clearly disrupted social connectedness, causing people to be lonelier [18,19].

Understanding loneliness is important for its connection with mental and physical health and the consequent economic outcomes. Social isolation and the feeling of loneliness lead to feelings of continuous stress and depression, creating physiological changes connected to the immune system and inflammatory responses, which further exacerbate physical and mental health conditions [13,15,16,20,21]. Previous studies also provide evidence that loneliness is associated with mental health conditions such as chronic stress [22]; anxiety and anger [23]; depression and neuroticism [12,13,24]; lower cognition, dementia, and Alzheimer’s disease [25,26,27]; and so on. Loneliness is associated with physical health conditions such as higher inflammation and fatigue [28,29], higher blood pressure [30], cardiovascular disease [30,31], and so on. The American Psychological Association [17] warned that the risk exposure of loneliness was greater than that of obesity and the impact was growing and would continue to grow in the future. In addition to the effect on physical and mental health, loneliness has consequences for people’s role as a social entity. When a person remains in a prolonged state of loneliness, negative social attitudes tend to develop such as shyness, negative moods, fear of negative evaluation, lower social skills, and so on. The greater negative social expectations in lonely persons can lead to the development of distrust, hostility, and intolerance [12].

The COVID-19 pandemic affected the individual, community, and social life of people such that their potential loneliness must be observed with due emphasis. The government-imposed restrictive measure such as the state of emergency in Japan, which was imposed during the early stage of the pandemic and continued throughout the year, had affected peoples’ usual personal and social lives. Although health authorities asked populations to maintain physical and social distancing, this measure is likely to exacerbate loneliness because social isolation and loneliness tend to occur together [32,33]. It has already been evident that the pandemic and its mitigating measures have increased loneliness among both younger and older people [1,3,5,6,7]. However, the contributing factors to the loneliness of younger and older people should be discussed carefully because social expectations and social networks might have differing effect on their loneliness [2,3,5,6]. For example, Weissbourd et al. [5] argue that changes in social network contribute differently to younger and older peoples’ loneliness. It appears that social isolation impacts more on younger peoples’ social network and older peoples’ cognitive and health issues [5,34]. Victor and Sullivan [35] argue that loneliness among older people is determined not only by individual factors but also by factors related to community and society. This finding shows that older peoples’ lifestyle and living conditions should be carefully thought before imposing social distancing measures. Older people generally depend on family members for assistance in performing daily activities or on institutional long-term care at home or in old care homes. While social distancing is important to limit the spread of viral infection, the health requirement can affect the receipt of care and sense of social connectedness, which could ultimately affect the physical and mental health of older people negatively. The consequence could be more serious for those with pre-existing loneliness and mental conditions.

Despite being an important public health issue, there are no longitudinal studies on how the pandemic influenced loneliness among older and younger people in Japan. To fill this gap, this study investigates the conditions of loneliness before and during the pandemic among older people and compares them with their younger counterparts in Japan to determine whether older people suffered more from loneliness during the pandemic. Additionally, we investigate the socio-demographic and psychological factors that led to loneliness during this pandemic. We hypothesize that older people became lonelier due to maintaining social distancing, enhanced precaution, lack of health and family care, fear, and anxiety.

## 2. Materials and Methods

### 2.1. Participants and Instruments

This study uses panel data from the Household Behavioral and Financial Survey funded by Hiroshima University. Nikkei Research, a leading research company in Japan, conducted the online survey. Nikkei Research has one of the largest databases in Japan, which includes a representative population from many socio-economic backgrounds. The first wave of the online survey was conducted at the outset of the COVID-19 pandemic from 20 to 25 February 2020. The second wave of the survey was conducted a year after the first wave from 19 to 26 February 2021 and targeted individuals who responded to the 2020 survey. The survey was conducted following a random sampling procedure while maintaining the representativeness of sample in both waves. All prospective participants were approached through online and they agreed to participate in the survey. Data were collected through questionnaires sent to each participant. The questionnaire included dichotomous, multiple, and scaling questions on demographic, socio-economic, and psychological characteristics and preferences of the prospective participants. The minimum age of the prospective participants was 20 years. The dataset had 17,463 and 6103 total observations in 2020 and 2021, respectively.

Data on loneliness, marital status, living status, employment status, household income, household assets, current health status, anxiety, feelings of depression, financial satisfaction, and future orientation were available in both 2020 and 2021 waves. However, data on gender, age, education, children in the household, living in rural areas, and financial literacy were available only in the 2020 wave. The inclusion criteria for this study were that minimum age of respondents had to be at least 20 years at the time of the survey and they have to answer all the questions included in this study. Thus, we had to exclude several observations due to missing socio-economic data such as household income, household assets, and financial literacy. Our final sample consists of 4253 respondents.

### 2.2. Ethical Statement and Conflict of Interest

This is a socio-economic study, which does not involve any invasiveness nor identifiable human aspects. Thus, the ethical review and approval were not necessary according to institutional requirements (the ethics committee of Hiroshima University, Higashihiroshima, Japan). However, all participants were informed about the purpose of the study before the survey and they agreed to take part in it. We also declare that there is no conflict of interest.

### 2.3. Variable Definitions

Loneliness is the main variable of interest, which we also use as the dependent variable in the regression analysis. We primarily followed the UCLA methodology to measure loneliness [36]. We measured loneliness as a binary variable where 1 indicates that the respondents are lonely and 0 otherwise. Respondents are classified as lonely if they responded that they felt a lack of companionship, left out, and isolated often or some of the time. To check the robustness of our results, we use a more direct alternative question to measure loneliness: “how often do you feel lonely?” Participants responded on a five-point scale ranging from often or always to never. The alternative measure of loneliness is also binary, where 1 indicates the respondent is lonely, and 0 otherwise. Respondents are classified as lonely if they reported feeling lonely always or often, some of the time, and occasionally.

As explanatory variables, we include gender, age, education, marital status, living status, living in a rural area, employment status, household income, and household assets as demographic and socio-economic variables. We also include financial literacy as a proxy for rational decision-making ability related to savings, investment, and health-related behaviors [37,38,39,40,41,42]. Furthermore, we include subjective health status, feelings of depression, future anxiety, financial satisfaction, and myopic view of the future to account for respondents’ subjective psychological and health concerns. Table 1 provides the definitions of all the variables.

### 2.4. Statistical Analysis

We use descriptive statistics to show the distribution of loneliness of the respondents and their demographic, socio-economic, and psychological characteristics. Moreover, we use a mean-comparison test to compare loneliness before and during the pandemic. In this study, loneliness before the pandemic means loneliness among respondents measured from data of the February, 2020 wave while loneliness during the pandemic means loneliness among respondents measured from data of the February, 2021 wave. Although the first case of the COVID-19 was detected in January 2020 in Japan, we labelled loneliness measured from the February, 2020 wave as “loneliness before pandemic” because the virus did not spread much during the time of the data collection. Moreover, the WHO declared the COVID-19 outbreak a global pandemic on 11 March 2020, which is after the first wave of the survey was conducted. Thus, we believe the loneliness among respondents and socio-economic conditions during February, 2020 largely reflect the situation when the COVID-19 was not considered a pandemic in Japan. Finally, we examine the association between loneliness and respondents’ demographic, socio-economic, psychological, and health-related factors using Equation (1).

(1) $$Y_{i1}=f\left(X_{i},\varepsilon_{i}\right)$$

where $Y_{1}$ is loneliness, X is a vector of individual characteristics, and ε is the error term. We use a logit regression to estimate Equation (1), as the dependent variable is binary. Because a logit model is used to model the probability of an event falling into one of the specified categories, we have used this model to predict loneliness against a set of socio-economic and psychological variables.

As the explanatory variables are potentially multicollinear, we conducted correlation and multicollinearity tests (results are unreported here to save space but are available upon request). The correlation matrix shows a weak relationship between the explanatory variables (lower than 0.70). In addition, the variance inflation factors of the explanatory variables are below 10, indicating that multicollinearity is not significant in the model.

Equation (2) provides the full model specifications of Equation (1):

(2) $$\begin{array}{cc} Loneliness_{i}= & \beta_{0}\;+\beta_{1}male_{i}+\beta_{2}age_{i}+\beta_{3}married_{i}+\beta_{4}children_{i}+\beta_{5}live\;alone_{i}\\ & +\beta_{6}living\;in\;rural\;area_{i}+\beta_{7}education_{i}+\beta_{8}employed_{i}+\beta_{9}household\;income_{i}\\ & +\beta_{10}household\;assets_{i}+\beta_{11}financial\;literacy_{i}+\beta_{12}subjective\;health_{i}\\ & +\beta_{13}future\;anxiety_{i}+\beta_{14}financial\;satisfaction_{i}+\beta_{15}depression_{i}\\ & +\beta_{16}myopic\;view\;of\;the\;future_{i}+\varepsilon_{i} \end{array}$$

## 3. Results

### 3.1. Descriptive Statistics

Table 2 provides the descriptive statistics of the main variables. The mean loneliness scores following the UCLA measure were 0.70 (SD = 0.46) and 0.74 (SD = 0.44) in 2020 and 2021, respectively, while those following the direct measure were 0.57 (SD = 0.50) and 0.59 (SD = 0.49) in 2020 and 2021, respectively. For the demographic variables, about 65.44% of the sample are men and the average age is 50.32 years. For household status, 66.05% are married, 57.11% have children, 20.15% live alone, and 58.15% (SD = 49.34%) do not live in Tokyo special wards nor government designated city areas. For socio-economic status, respondents attained 14.97 years (SD = 2.11 years) of education, about 63.81% are currently employed, have annual household incomes of JPY 6.31 million (SD = JPY 4.05 million), and have a balance of JPY 19.80 million (SD = JPY 29.10 million) in household assets. Moreover, respondents have an average financial literacy score of 0.65 (SD = 0.36); that is, they can answer nearly 2 out of the 3 financial literacy questions correctly. For psychological and health issues, respondents rated their subjective health status, anxiety about future life, satisfaction with financial status, feelings of depression, and myopic view about the future at 3.25, 3.71, 2.74, 2.98, and 2.69, respectively, out of 5.

Table 3 compares loneliness in 2020 and 2021 for older (more than or equal to 65 years of age) and younger (less than 65 years of age) respondents. For older respondents, the loneliness scores following the UCLA measure were 0.56 (SD = 0.50) and 0.60 (SD = 0.49) in 2020 and 2021, respectively, while those following the direct measure were 0.36 (SD = 0.48) and 0.40 (SD = 0.49) in 2020 and 2021, respectively. For younger respondents, the loneliness scores following the UCLA measure were 0.73 (SD = 0.44) and 0.77 (SD = 0.42) in 2020 and 2021, respectively, while those following the direct measure were 0.61 (SD = 0.49) and 0.63 (SD = 0.48) in 2020 and 2021, respectively. We can see a significant increase in loneliness both among older and younger people in Japan according to both measures of loneliness.

### 3.2. Regression Results

We use two sets of logit regression models for the two measures of loneliness to determine the factors that explain loneliness before (2020) and during the COVID-19 pandemic (2021). The two measures of loneliness are the dependent variables for two sets of models. The explanatory variables remain the same in all regression models. We investigate loneliness for the full sample as well as the older and younger sub-samples. The LR chi^2^ values of all models are statistically significant, suggesting good fit of the models.

Table 4 shows the regression results for the loneliness of the full sample before and during the pandemic. The results suggest that respondents with younger age, higher balance of household assets, subjective health conditions, lower satisfaction about current financial condition, feelings of depression, and a myopic view about the future were lonely before the pandemic while respondents with younger age, the status of living alone, less household income, subjective health conditions, feelings of depression, and a myopic view of future were lonely during the pandemic. However, the evidence of loneliness before the pandemic among respondents who are males, not currently married, live alone, and have less household income is not robust. Similarly, the evidence of loneliness during the pandemic among respondents who have higher balance of household assets, anxiety about future life, and less satisfaction about current financial condition is not robust.

As loneliness among older people increased significantly during the pandemic, we conduct a regression analysis for sub-samples of older and younger respondents. Table 5 shows the logit regression coefficients for the UCLA and direct measures in 2020 and 2021 for the older sub-sample. The results suggest that respondents who are relatively younger, live alone, and have feelings of depression were lonely before the pandemic while respondents who are relatively younger, have subjective health conditions, feelings of depression, and myopic view of future were lonely during the pandemic. However, the evidence of loneliness before the pandemic among respondents who are males, have lower household income, higher balance of household assets, and subjective health conditions is not robust. Similarly, the evidence of loneliness during the pandemic among respondents who are males, live alone, not currently employed, anxious about future life, and less satisfied about current financial condition is not robust.

Table 6 reports the logit regression coefficients with both measures in 2020 and 2021 for the younger sub-sample. The results suggest that respondents who are younger, not currently married, have subjective health conditions, less satisfied with financial condition, have feelings of depression, and myopic view of future were lonely before the pandemic, while respondents who have less household income, subjective health conditions, less financial satisfaction, feelings of depression, and myopic view of future were lonely during the pandemic. However, the evidence of loneliness before the pandemic among respondents who are males, live alone, have less household income, and higher balance of household assets is not robust. Similarly, the evidence of loneliness during the pandemic among respondents who are younger, live alone, and anxious about future life is not robust.

As the mean comparison test shows that loneliness intensified considerably during the ongoing pandemic, we investigate the demographic, socio-economic, and psychological factors associated with loneliness for respondents who became lonely during the pandemic but were not lonely before. In the logit regression models, we use loneliness as a binary variable, where 1 indicates respondents who became lonely during the pandemic but were not lonely before, and 0 otherwise. The explanatory variables include male, age, married, having children, living alone, living in a rural area, education, employed, log of household income and assets, financial literacy, health status, anxiety about future life, satisfaction with current financial condition, feelings of depression, and myopic view of the future during the pandemic. The regression results in Table 7 show that older respondents who have more household income and satisfaction about current financial condition became lonely during the pandemic. On the other hand, younger respondents who are females, currently married, living alone, and are not depressive became lonely during the pandemic.

## 4. Discussion

### 4.1. Loneliness among Older and Younger People

We sought to explain two interrelated issues regarding loneliness during the COVID-19 pandemic. First, we studied whether loneliness intensified during the pandemic, and if so, whether older people were affected more than younger people. Second, we investigated the factors that could explain loneliness before and during the pandemic, and what made people lonely during the pandemic who were not lonely before.

Regarding the first issue, we found that loneliness among both younger and older people intensified significantly during the pandemic. Loneliness among younger people was pervasive and higher than that of older people both before and during the pandemic. However, the magnitude of increase in loneliness during the pandemic is slightly higher for older people. Older people are seen respectfully in Japanese families, where family members tend to take care of them [43,44]. Older people feel accomplished and happy living with their family members. However, all these factors that provided older people with social connection were severely disturbed during the pandemic due to health safety measures such as quarantines, social distancing, avoiding social gatherings, and avoiding going out unnecessarily [32,45,46,47]. Moreover, anxiety about health and future life, lack of the required long-term care services, and living under stress contributed to feel lonely [48]. While the concurrent literature provides evidence on intensifying loneliness during the pandemic [7,49], the populations affected differ to some extent. Some results provide no clear indication of an increase in loneliness overall [50], increase in loneliness among younger adults [7,51], or among older adults [3,6]. Our result is consistent with previous studies as we provide evidence of increasing loneliness among older as well as younger people.

### 4.2. Factors Explaining Loneliness among Older and Younger People before and during the Pandemic

While investigating the factors explaining loneliness before and during the pandemic, we found that subjective health condition, feelings of depression, and age are the most robust and consistent factors behind loneliness for both older and younger people. People with lower subjective health status suffer from loneliness because it impairs their social connectedness. Positive engagement in a social network facilitates peoples’ mental soundness and helps them avoid loneliness [52,53]. The findings of Savikko et al. [54] and Tilvis, Jolkkonen, and Strandberg [55] that peoples’ actual and subjective health status are significant predictors of loneliness support our findings and argument. Subjective health status among elderly people has added significance because of their need for long-term care. Elias [56] and Drageset, Kirkevold, and Espehaug [57] find that elderly people who need long-term care might develop loneliness even if they receive care from family members or from caregiving institutions.

Besides subjective health condition, feeling of depression appears to be another strong predictor of loneliness among older and younger people, both before and during the pandemic. Depression is a mood disorder that leads to disinterest in regular activities. Eventually, people with depression become less sociable, loose contact with family and friends, and develop loneliness. Tilvis, Jolkkonen, and Strandberg [55], Singh and Misra [58], Kato et al. [59] and Watts [60] show that depression is a predictor of loneliness. Several studies document increased levels of depression during the COVID-19 pandemic [61,62,63]. With sadness all around and anxiety about health and livelihood, the pandemic and the associated social distancing measures intensified the feelings of depression, leading to loneliness [64].

Globally, loneliness among younger people has been a well-evident phenomenon, which seems to be intensified during the pandemic [7,51]. We also provide evidence of increasing loneliness among younger people in Japan. We argue that lack of support from families and friends due the changing social structure could be the reason behind loneliness among younger people. Sometimes, socio-economic conditions, pressure in the workplace, and new roles in life also contribute to their loneliness. Moreover, excessive use of digital devices and online social media might hamper their social life.

We observe differences in the covariates of loneliness between older and younger people in some cases too. First, myopic view of the future was a predictor of loneliness among older people during the pandemic, but was significant both before and during the pandemic for younger people. Since future orientation shapes peoples’ outlook toward the future through social engagement [65,66], people with myopic future orientation are less likely to place due importance on these social aspects and are thus more likely to be lonely. Second, lower household income and less satisfaction about current financial condition seem to shape younger people’s loneliness more than their older counterparts. Third, living alone is a significant predictor of loneliness among older people. When people live alone, their desire for social relations is severely compromised and make them lonely [11,12,13]. Fourth, younger people who are currently not married were found to be lonely before the pandemic.

Our findings show that many people became lonely during the pandemic who were not lonely before. Older people who have more household income and are financially satisfied became lonely during the pandemic. On the other hand, younger respondents who are female, currently married, living alone, and were not depressed before became lonely during the pandemic. Our finding that female became lonely during the pandemic is consistent with previous studies [67,68]. Females may have become lonelier during the pandemic for several reasons. Previous studies find that compared to males, females are more expressive of their feelings and emotions, value human relationships more, tend to build more social networks, and live longer, exposing them to widowhood and other losses [54,67,69,70,71]. The social isolation and distancing measures during the pandemic affected social relationships and normal activities to such an extent that females might have faced difficulty expressing their feelings and maintaining human relationship, making them lonely. Moreover, younger people who are currently married and do not have feelings of depression also became lonely during the pandemic. These findings show that people who are not likely to be lonely in normal situation became lonely during the pandemic due to changing social relationships and the pandemic measures. However, younger people who are living alone also became lonely during the pandemic. Why older people with higher income and financial satisfaction became lonely during the pandemic requires investigation as well, because people in the higher socio-economic statuses are generally less likely to be lonely [72,73]. People with more income and financial satisfaction, who are apparently in a higher socio-economic status, might have witnessed changes in their lifestyles and social interactions due to changing social structure during the pandemic.

### 4.3. Strengths and Limitations

The strength of this study is that it is one of the few studies that compares loneliness between older and younger people in Japan before and during the pandemic. The study further identifies the critical factors that increase older and younger people’s vulnerability to loneliness. These factors will be useful in targeting the appropriate population for strategies to reduce and prevent loneliness as well as the resulting consequences.

This study has some limitations that should be considered while interpreting the results. First, as we use data from an internet survey, we cannot rule out a difference in the household income distribution between our sample and overall Japanese population. Second, we had to exclude several observations due to missing values on household income, household assets, and financial literacy. Third, our data does not include information on whether participants suffered from the COVID-19 virus or experienced any grief during the pandemic. We cannot ignore the possibility that these issues could have influenced results of this study. Nevertheless, our study provides robust and consistent evidence that a significant number of older and younger people became lonely in Japan and that the vulnerability to loneliness increases with specific socio-economic issues.

## 5. Conclusions

This study investigated loneliness among older and younger people before and during the COVID-19 pandemic in Japan. We provide evidence on the increasing magnitude of loneliness among both older and younger people in Japan during the pandemic. However, the magnitude of increase in loneliness is slightly higher for older people compared to their younger counterparts. People with younger ages, poor subjective health status and feelings of depression were lonely before and during the pandemic. Household income and financial satisfaction are found to be associated with loneliness among older people during the pandemic who were not lonely before while gender, marital status, living status, and feelings of depression are found to be associated with loneliness among younger people who were not lonely before. We suggest that needs-based special social networks should be established for those with a higher likelihood of becoming lonely during the pandemic. Authorities can publicize and patronize the formation of such special social networks through educational institutions, private and public offices, and social groups. Moreover, authorities could introduce psychological intervention programs such as mindfulness therapy, cognitive enhancement programs, and so on, through online and digital platforms for people suffering from acute loneliness.

## Figures and Tables

**Table 1:** Variable definitions.

Variable	Definition
**Dependent variable**	
Loneliness	The extent to which respondents feel loneliness according to the UCLA methodology. The questions asked to measure respondents’ loneliness were “How often do you feel a lack of companionship,” “How often do you feel left out,” and “How often do you feel isolated from others.” The options to respond to these questions were “Hardly ever or never,” “Some of the time,” and “Often.” Loneliness is a binary variable where 1 indicates having feelings of loneliness some of the time or often, and 0 otherwise. As an alternative, we measured loneliness using the question “How often do you feel lonely” (1 being never and 5 being often or always). This alternative measure of loneliness as a binary variable where 1 indicates feeling loneliness often or always, some of the time, and occasionally, and 0 otherwise.
**Explanatory variables**	
Male *	Binary variable: 1 = Male and 0 = Female
Age *	Continuous variable: Respondent’s age
Married	Binary variable: 1 = Currently married and 0 = Otherwise
Children *	Binary variable: 1 = Have child/children and 0 = Otherwise
Live alone	Binary variable: 1 = Living alone and 0 = Otherwise
Living in rural area	Binary variable: 1 = Living in rural areas (not Tokyo special wards or government designated city areas) and 0 = Otherwise
Education *	Continuous variable: Years of education
Employed	Binary variable: 1 = Respondent is employed and 0 = otherwise
Household income	Continuous variable: Annual earned income before taxes and with bonuses of the entire household in 2020 (unit: JPY)
Log of household income	Log of household income
Household assets	Continuous variable: Balance of financial assets (savings, stocks, bonds, insurance, etc.) of the entire household (unit: JPY)
Log of household assets	Log of household assets
Financial literacy *	Continuous variable: Average correct answers to three financial literacy questions
Subjective health status	Ordinal variable: 1 = It does not hold true at all for you; 2 = It is not so true for you; 3 = Neither true nor not true; 4 = It is rather true for you; 5 = It is particularly true for you, for the statement, “I am now healthy and was generally healthy in the last one year.”
Future anxiety	Ordinal variable: 1 = It does not hold true at all for you; 2 = It is not so true for you; 3 = Neither true nor not true; 4 = It is rather true for you; 5 = It is particularly true for you, for the statement, “I have anxieties about my life after I am 65 years old (for those who are already aged 65 or above, ‘life in the future’).”
Financial satisfaction	Ordinal variable: 1 = Completely disagree; 2 = Disagree; 3 = Neither agree nor disagree; 4 = Agree; 5 = Completely agree, for the statement, “Since the future is uncertain, it is a waste to think about it.” I am happy with my financial status.”
Feeling of depression	Ordinal variable: 1 = It does not hold true at all for you; 2 = It is not so true for you; 3 = Neither true nor not true; 4 = It is rather true for you; 5 = It is particularly true for you, for the statement, “I often feel depressed or felt depressed in the last one year.”
Myopic view of the future	Ordinal variable: 1 = Completely disagree; 2 = Disagree; 3 = Neither agree nor disagree; 4 = Agree; 5 = Completely agree, for the statement, “Since the future is uncertain, it is a waste to think about it.”

Note: * indicates data from the 2020 wave.

**Table 2:** Descriptive Statistics.

Variable	Obs.	Mean	Std. Dev.	Min	Max
Loneliness 2020 (UCLA Measure)	4253	0.6981	0.4591	0	1
Loneliness 2021 (UCLA Measure)	4253	0.7364	0.4406	0	1
Loneliness 2020 (Direct Measure)	4253	0.5648	0.4958	0	1
Loneliness 2021 (Direct Measure)	4253	0.5850	0.4928	0	1
Male	4253	0.6544	0.4756	0	1
Age	4253	50.3184	13.8258	21	86
Married	4253	0.6605	0.4736	0	1
Children	4253	0.5711	0.4950	0	1
Living alone	4253	0.2015	0.4012	0	1
Living in rural area	4253	0.5815	0.4934	0	1
Education	4253	14.9697	2.1129	9	21
Employed	4253	0.6381	0.4806	0	1
Household income	4253	6.3085	4.0456	0.50	21.00
Log of HHIncome	4253	15.4271	0.7598	13.12	16.86
Household assets	4253	19.8000	29.1000	1.25	125.00
Log of HHAssets	4253	15.8515	1.4297	14.04	18.64
Finliteracy	4253	0.6524	0.3568	0	1
Healthy	4253	3.2445	1.0878	1	5
Anxiety	4253	3.7129	1.1380	1	5
Finsatisfaction	4253	2.7437	1.1153	1	5
Depression	4253	2.9758	1.2171	1	5
Myopic view	4253	2.6852	1.0174	1	5

**Table 3:** Comparison of loneliness before and during the COVID-19 pandemic.

Variable	Mean	Std. Error	Obs.
Full sample			
Loneliness 2020 (UCLA Measure)	0.6981	0.0070	4253
Loneliness 2021 (UCLA Measure)	0.7364	0.0068	4253
Difference	0.0383 (3.93) ***		
Loneliness 2020 (Direct Measure)	0.5648	0.0076	4253
Loneliness 2021 (Direct Measure)	0.5850	0.0076	4253
Difference	0.0202 (1.89) *		
Older sub-sample			
Loneliness 2020 (UCLA Measure)	0.5586	0.0179	768
Loneliness 2021 (UCLA Measure)	0.6016	0.0177	768
Difference	0.0430 (2.50) **		
Loneliness 2020 (Direct Measure)	0.3607	0.0173	768
Loneliness 2021 (Direct Measure)	0.3958	0.0177	768
Difference	0.0352 (2.07) **		
Younger sub-sample			
Loneliness 2020 (UCLA Measure)	0.7288	0.0075	3485
Loneliness 2021 (UCLA Measure)	0.7661	0.0072	3485
Difference	0.0373 (5.02) ***		
Loneliness 2020 (Direct Measure)	0.6098	0.0083	3485
Loneliness 2021 (Direct Measure)	0.6267	0.0082	3485
Difference	0.0169 (2.04) **		

Note: *** *p* < 0.01, ** *p* < 0.05, * *p* < 0.10.

**Table 4:** Logit regression analysis for loneliness before and during the COVID-19 pandemic.

Variable	UCLA Measure		Direct Measure	
	2020	2021	2020	2021
Male	0.4065 (4.04) ***	0.1245 (1.28)	0.0668 (0.71)	−0.0128 (−0.14)
Age	−0.0223 (−5.89) ***	−0.0174 (−4.90) ***	−0.0267 (−7.49) ***	−0.0219 (−6.63) ***
Married	−0.1670 (−1.23)	0.0194 (0.15)	−0.3144 (−2.54) **	−0.1652 (−1.43)
Children	−0.1249 (−1.21)	−0.1585 (1.60)	0.1350 (1.39)	0.0549 (0.60)
Living alone	0.0304 (0.20)	0.2795 (1.99) **	0.2934 (2.20) **	0.4077 (3.24) ***
Living in rural area	0.0864 (1.09)	0.0373 (0.49)	0.0617 (0.82)	0.1013 (1.42)
Education	0.0002 (0.01)	0.0105 (0.53)	0.0071 (0.37)	0.0279 (1.54)
Employed	−0.0836 (−0.82)	−0.0586 (−0.60)	0.1377 (1.43)	0.1211 (1.35)
Log of HHIncome	−0.2364 (−3.28) ***	−0.1731 (−2.56) ***	−0.0316 (−0.48)	−0.1064 (−1.73) *
Log of HHasset	0.0758 (2.21) **	0.0348 (1.04)	0.0877 (2.71) ***	0.0556 (1.77) *
Finliteracy	−0.0129 (−0.10)	0.0139 (0.12)	0.0792 (0.67)	−0.1394 (−1.29)
Healthy	−0.1857 (−4.78) ***	−0.2608 (−6.93) ***	−0.2395 (−6.46) ***	−0.2192 (−6.27) ***
Anxiety	0.0513 (1.37)	0.1318 (3.45) ***	−0.0297 (−0.82)	0.0592 (1.62)
Finsatisfaction	−0.1262 (−2.94) ***	−0.0547 (−1.32)	−0.1138 (−2.79) ***	−0.1098 (−2.85) ***
Depression	0.5459 (13.60) ***	0.4528 (12.41) ***	0.6386 (16.68) ***	0.6156 (18.03) ***
Myopic view	0.1169 (2.84) ***	0.0942 (2.48) **	0.0906 (2.32) **	0.0994 (2.77) ***
_Cons	3.3667 (3.10) ***	2.8953 (2.86) ***	−0.4218 (−0.43)	0.5325 (0.58)
Obs.	3755	4253	3755	4253
LR Chi2	586.32 ***	553.95 ***	766.99 ***	906.25 ***
Pseudo R2	0.1275	0.1129	0.1489	0.1570
Log likelihood	−2006.9567	−2176.0138	−2192.3659	−2433.0781

Note: *z* values in parentheses; *** *p* < 0.01, ** *p* < 0.05, * *p* < 0.10.

**Table 5:** Logit regression analysis for loneliness before and during the COVID-19 pandemic for the older sub-sample.

Variable	UCLA Measure		Direct Measure	
	2020	2021	2020	2021
Male	0.9722 (3.57) ***	0.8446 (3.56) ***	0.3935 (1.40)	0.2780 (1.13)
Age	−0.0614 (−2.61) ***	−0.0583 (−2.94) ***	−0.0567 (−2.25) **	−0.0391 (−1.89) *
Married	0.6456 (1.35)	−0.0841 (−0.23)	0.5280 (1.04)	−0.0812 (−0.22)
Children	−0.0436 (−0.15)	−0.1757 (−0.68)	0.4891 (1.54)	−0.2640 (−1.04)
Living alone	0.9984 (1.84) *	0.6555 (1.56)	1.4129 (2.58) ***	0.7995 (1.94) *
Living in rural area	0.1445 (0.78)	−0.0864 (−0.52)	−0.1201 (−0.63)	0.0238 (0.14)
Education	−0.0368 (−0.79)	0.0231 (0.54)	0.0653 (1.33)	0.0419 (0.97)
Employed	−0.2459 (−1.09)	−0.4706 (−2.39) **	−0.2794 (−1.19)	0.0155 (0.08)
Log of HHIncome	−0.6673 (−3.52) ***	−0.1370 (−0.86)	−0.1359 (−0.74)	0.0028 (0.02)
Log of HHasset	0.1309 (1.63)	0.0253 (0.35)	0.2024 (2.42) **	0.0792 (1.07)
Finliteracy	−0.1331 (−0.39)	−0.0082 (−0.03)	0.2668 (0.76)	−0.0534 (−0.19)
Healthy	−0.0765 (−0.85)	−0.2567 (−3.29) ***	−0.1873 (−1.98) **	−0.1716 (−2.17) **
Anxiety	0.0418 (0.44)	0.2120 (2.18) **	0.0031 (0.03)	0.0036 (0.04)
Finsatisfaction	0.0556 (0.48)	0.1670 (1.53)	−0.0883 (−0.74)	−0.1974 (−1.79) *
Depression	0.6808 (6.58) ***	0.3882 (4.66) ***	0.7203 (6.91) ***	0.6509 (7.60) ***
Myopic view	0.1056 (1.03)	0.1457 (1.82) *	0.1093 (1.02)	0.1782 (2.13) **
_Cons	9.7889 (3.08) ***	3.7981 (1.39)	−1.6299 (−0.51)	−0.6951 (−0.25)
Obs.	631	768	631	768
LR Chi^2^	129.86 ***	105.42 ***	118.67 ***	140.59 ***
Pseudo R^2^	0.1495	0.1021	0.1459	0.1364
Log likelihood	−369.4942	−463.6727	−347.4110	−445.2509

Note: *z* values in parentheses; *** *p* < 0.01, ** *p* < 0.05, * *p* < 0.10.

**Table 6:** Logit regression analysis for loneliness before and during the COVID-19 pandemic for the younger sub-sample.

Variable	UCLA Measure		Direct Measure	
	2020	2021	2020	2021
Male	0.3221 (2.91) ***	−0.0403 (−0.37)	0.0064 (0.06)	−0.0480 (−0.49)
Age	−0.0135 (−2.87) ***	−0.0072 (−1.56)	−0.0178 (−4.12) ***	−0.0173 (−4.16) ***
Married	−0.2609 (−1.80) *	0.0341 (0.25)	−0.3703 (−2.84) ***	−0.1722 (−1.39)
Children	−0.1204 (−1.08)	−0.1458 (−1.33)	0.1209 (1.16)	0.1049 (1.07)
Living alone	−0.0659 (−0.42)	0.2179 (1.44)	0.2320 (1.65) *	0.3633 (2.71) ***
Living in rural area	0.0838 (0.95)	0.0704 (0.81)	0.1027 (1.25)	0.1184 (1.50)
Education	0.0061 (0.26)	0.0066 (0.30)	−0.0033 (−0.16)	0.0265 (1.32)
Employed	−0.0959 (−0.78)	0.0292 (0.25)	0.1697 (1.52)	0.1119 (1.06)
Log of HHIncome	−0.1712 (−2.17) **	−0.1983 (−2.60) ***	−0.0197 (−0.28)	−0.1351 (−2.01) **
Log of HHasset	0.0617 (1.60)	0.0386 (1.00)	0.0620 (1.75) *	0.0538 (1.54)
Finliteracy	−0.0039 (−0.03)	0.0206 (0.16)	0.0616 (0.48)	−0.1570 (−1.33)
Healthy	−0.2007 (−4.60) ***	−0.2539 (−5.84) ***	−0.2434 (−5.98) ***	−0.2245 (−5.71) ***
Anxiety	0.0558 (1.36)	0.1206 (2.85) ***	−0.0339 (−0.86)	0.0649 (1.64)
Finsatisfaction	−0.1560 (−3.34) ***	−0.0941 (−2.07) **	−0.1086 (−2.49) **	−0.0959 (−2.32) **
Depression	0.5229 (11.88) ***	0.4743 (11.54) ***	0.6247 (15.08) ***	0.6126 (16.33) ***
Myopic view	0.1182 (2.60) ***	0.0828 (1.90) *	0.0845 (2.00) **	0.0842 (2.11) **
_Cons	2.4164 (2.05) **	2.9502 (2.63) ***	−0.2982 (−0.28)	0.8559 (0.86)
Obs.	3124	3485	3124	3485
LR Chi^2^	412.21 ***	400.99 ***	535.38 ***	643.24 ***
Pseudo R^2^	0.1128	0.1058	0.1276	0.1397
Log likelihood	−1621.4628	−1694.9911	−1830.8421	−1980.9065

Note: *z* values in parentheses; *** *p* < 0.01, ** *p* < 0.05, * *p* < 0.10.

**Table 7:** Logit regression analysis for loneliness among respondents during the COVID-19 pandemic who were not lonely before.

Variable	Full Sample	Older Sub-Sample	Younger Sub-Sample
Male	−0.2951 (−2.45) ***	0.1743 (0.53)	−0.4044 (−3.04) ***
Age	0.0002 (0.05)	−0.0351 (−1.27)	−0.0044 (−0.76)
Married	0.5000 (2.93) ***	−0.1026 (−0.21)	0.5266 (2.85) ***
Children	−0.0314 (−0.25)	−0.2102 (−0.61)	−0.0029 (−0.02)
Living alone	0.3537 (1.94) *	−0.3165 (−0.55)	0.4082 (2.09) ***
Living in rural area	−0.0624 (−0.64)	−0.1591 (−0.72)	−0.0531 (−0.49)
Education	−0.0121 (−0.48)	−0.0294 (−0.51)	−0.0146 (−0.52)
Employed	−0.0397 (−0.32)	−0.2483 (−0.93)	0.0959 (0.67)
Log of HHIncome	0.1642 (1.89) *	0.5419 (2.41) **	0.1227 (1.28)
Log of HHasset	−0.0203 (−0.47)	−0.0674 (−0.69)	−0.0130 (−0.26)
Finliteracy	−0.1116 (−0.76)	0.4897 (1.23)	−0.1968 (−1.22)
Healthy	0.0524 (1.11)	0.1180 (1.10)	0.0338 (0.64)
Anxiety	0.0027 (0.05)	0.1545 (1.15)	−0.0083 (−0.15)
Finsatisfaction	0.0658 (1.25)	0.2912 (1.96) **	0.0417 (0.73)
Depression	−0.0500 (−1.11)	0.1586 (1.45)	−0.0915 (−1.84) *
Myopic view	−0.0071 (−0.15)	−0.0623 (−0.57)	0.0150 (0.28)
_Cons	−4.3271 (−3.32) ***	−8.2792 (−2.10) **	−3.3866 (−2.39) **
Obs.	4253	768	3485
LR Chi^2^	40.95 ***	20.49	43.47 ***
Pseudo R^2^	0.0132	0.0336	0.0174
Log likelihood	−1533.3514	−294.3110	−1226.3969

Note: *z* values in parentheses; *** *p* < 0.01, ** *p* < 0.05, * *p* < 0.10.

## Data Availability

Data are available on request.

## References

[1] Sweet J. The Loneliness Pandemic. https://harvardmagazine.com/2021/01/feature-the-loneliness-pandemic.

[2] Creese B., Khan Z., Henley W., O’Dwyer S., Corbett A., Da Silva M.V., Mills K., Wright N., Testad I., Aarsland D. (2021). Loneliness, physical activity, and mental health during COVID-19: A longitudinal analysis of depression and anxiety in adults over the age of 50 between 2015 and 2020. Int. Psychogeriatr..

[3] Seifert A., Hassler B. (2020). Impact of the COVID-19 pandemic on loneliness among older adults. Front. Sociol..

[4] Japan Times Older People in Japan Feeling Isolated amid Pandemic. https://www.japantimes.co.jp/news/2020/09/23/national/science-health/elderly-loneliness-coronavirus/.

[5] Weissbourd R., Batanova M., Lovison V., Torres E. Loneliness in America how the Pandemic Has Deepened an Epidemic of Loneliness and What We Can Do about it?. https://mcc.gse.harvard.edu/reports/loneliness-in-america.

[6] Davis M.R. (2020). Pandemic Has Created Loneliness Epidemic. AARP Foundation. https://www.aarp.org/home-family/friends-family/info-2020/isolation-survey-coronavirus.html.

[7] McGinty E.E., Presskreischer R., Han H., Barry C.L. (2020). Psychological distress and loneliness reported by US adults in 2018 and April 2020. JAMA.

[8] Koyama Y., Nawa N., Yamaoka Y., Nishimura H., Sonoda S., Kuramochi J., Miyazaki Y., Fujiwara T. (2021). Interplay between social isolation and loneliness and chronic systemic inflammation during the COVID-19 pandemic in Japan: Results from u-corona study. Brain Behav. Immun..

[9] Smith B.J., Lim M.H. (2020). How the COVID-19 pandemic is focusing attention on loneliness and social isolation. Public Health Res. Pract..

[10] Leigh-Hunt N., Bagguley D., Bash K., Turner V., Turnbull S., Valtorta N., Caan W. (2017). An overview of systematic reviews on the public health consequences of social isolation and loneliness. Public Health.

[11] Perlman D., Peplau L.A., Gilmour R., Duck S. (1981). Toward a social psychology of loneliness. Personal Relationships. 3: Personal Relationships in Disorder.

[12] Hawkley L., Baumeister R.F., Vohs K.D. (2007). Loneliness. Encyclopedia of Social Psychology.

[13] Cacioppo S., Grippo A.J., London S., Goossens L., Cacioppo J.T. (2015). Loneliness: Clinical import and interventions. Perspect. Psychol. Sci..

[14] Cacioppo J.T., Patrick W. (2008). Loneliness: Human Nature and the Need for Social Connection.

[15] Anderson G.O. (2010). Loneliness Among Older Adults: A National Survey of Adults 45+.

[16] Anderson G.O., Colette E.T. (2018). Loneliness and Social Connections: A National Survey of Adults 45 and Older.

[17] American Psychological Association So Lonely I Could Die. https://www.apa.org/news/press/releases/2017/08/lonely-die.

[18] AARP Foundation (2020). The Pandemic Effect: A Social Isolation Report.

[19] Fujiwara D., Dolan P., Lawton R., Behzadnejad F., Lagarde A., Maxwell C., Peytrignet S. The Wellbeing Costs of COVID-19 in the UK. https://www.jacobs.com/sites/default/files/2020-05/jacobs-wellbeing-costs-of-covid-19-uk.pdf.

[20] Cole S.W., Hawkley L.C., Arevalo J.M., Cacioppo J.T. (2011). Transcript origin analysis identifies antigen-presenting cells as primary targets of socially regulated gene expression in leukocytes. Proc. Natl. Acad. Sci. USA.

[21] Cacioppo J.T., Ernst J.M., Burleson M.H., McClintock M.K., Malarkey W.B., Hawkley L.C., Kowalewski R.B., Paulsen A., Hobson J.A., Hugdahl K. (2000). Lonely traits and concomitant physiological processes: The MacArthur social neuroscience studies. Int. J. Psychophysiol..

[22] Hawkley L.C., Burleson M.H., Berntson G.G., Cacioppo J.T. (2003). Loneliness in everyday life: Cardiovascular activity, psychosocial context, and health behaviors. J. Pers. Soc. Psychol..

[23] Cacioppo J.T., Hughes M.E., Waite L.J., Hawkley L.C., Thisted R.A. (2006). Loneliness as a specific risk factor for depressive symptoms: Cross-sectional and longitudinal analyses. Psychol. Aging.

[24] Erzen E., Çikrikci Ö. (2018). The effect of loneliness on depression: A meta-analysis. Int. J. Soc. Psychiatry.

[25] Sutin A.R., Stephan Y., Luchetti M., Terracciano A. (2018). Loneliness and risk of dementia. J. Gerontol. Ser. B.

[26] Shankar A., Hamer M., McMunn A., Steptoe A. (2013). Social isolation and loneliness: Relationships with cognitive function during 4 years of follow-up in the English longitudinal study of ageing. Psychosom. Med..

[27] Wilson R.S., Krueger K.R., Arnold S.E., Schneider J.A., Kelly J.F., Barnes L.L., Tang Y., Bennett D.A. (2007). Loneliness and risk of Alzheimer disease. Arch. Gen. Psychiatry.

[28] Jaremka L.M., Fagundes C.P., Glaser R., Bennett J.M., Malarkey W.B., Kiecolt-Glaser J.K. (2013). Loneliness predicts pain, depression, and fatigue: Understanding the role of immune dysregulation. Psychoneuroendocrinology.

[29] Luanaigh C.O., Lawlor B.A. (2008). Loneliness and the health of older people. Int. J. Geriatr. Psychiatry.

[30] Hawkley L.C., Thisted R.A., Masi C.M., Cacioppo J.T. (2010). Loneliness predicts increased blood pressure: 5-year cross-lagged analyses in middle-aged and older adults. Psychol. Aging.

[31] Caspi A., Harrington H., Moffitt T.E., Milne B.J., Poulton R. (2006). Socially isolated children 20 years later: Risk of cardiovascular disease. Arch. Pediatr. Adolesc. Med..

[32] Hwang T.J., Rabheru K., Peisah C., Reichman W., Ikeda M. (2020). Loneliness and social isolation during the COVID-19 pandemic. Int. Psychogeriatr..

[33] Banerjee D., Rai M. (2020). Social isolation in Covid-19: The impact of loneliness. Int. J. Soc. Psychiatry.

[34] Marczak J., Wittenberg R., Doetter L.F., Casanova G., Golinowska S., Guillen M., Rothgang H. (2019). Preventing social isolation and loneliness among older people. Eurohealth Obs..

[35] Victor C., Sullivan M.P., Twigg J., Martin W. (2015). Loneliness and Isolation. Handbook of Cultural Gerontology.

[36] Hughes M.E., Waite L.J., Hawkley L.C., Cacioppo J.T. (2004). A short scale for measuring loneliness in large surveys: Results from two population-based studies. Res. Aging.

[37] Kadoya Y., Khan M.S.R. (2018). Can financial literacy reduce anxiety about life in old age?. J. Risk Res..

[38] Kadoya Y., Khan M.S.R., Hamada T., Dominguez A. (2018). Financial literacy and anxiety about life in old age: Evidence from the USA. Rev. Econ. Househ..

[39] Khan M.S.R., Rabbani N., Kadoya Y. (2020). Is financial literacy associated with investment in financial markets in the United States?. Sustainability.

[40] Khan M.S.R., Putthinun P., Watanapongvanich S., Yuktadatta P., Uddin M.A., Kadoya Y. (2021). Do financial literacy and financial education influence smoking behavior in the United States?. Int. J. Environ. Res. Public Health.

[41] Watanapongvanich S., Khan M.S.R., Putthinun P., Ono S., Kadoya Y. (2020). Financial literacy, financial education, and smoking behavior: Evidence from Japan. Front. Public Health.

[42] Watanapongvanich S., Binnagan P., Putthinun P., Khan M.S.R., Kadoya Y. (2021). Financial literacy and gambling behavior: Evidence from Japan. J. Gambl. Stud..

[43] Yamaguchi S., Cohen S.R., Uza M. (2016). Family caregiving in Japan: The influence of cultural constructs in the care of adults with cancer. J. Fam. Nurs..

[44] Hanaoka C., Norton E.C. (2008). Informal and formal care for elderly persons: How adult children’s characteristics affect the use of formal care in Japan. Soc. Sci. Med..

[45] Hajek A., König H.H. (2021). Social isolation and loneliness of older adults in times of the COVID-19 pandemic: Can use of online social media sites and video chats assist in mitigating social isolation and loneliness?. Gerontology.

[46] American Psychological Association COVID-19 Isn’t Just a Danger to Older People’s Physical Health. https://www.apa.org/news/apa/2020/03/covid-19-danger-physical-health.

[47] Miller G. (2020). Social distancing prevents infections, but it can have unintended consequences. Science.

[48] Nelson B.W., Pettitt A.K., Flannery J.E., Allen N.B. (2020). Rapid assessment of psychological and epidemiological correlates of COVID-19 concern, financial strain, and health-related behavior change in a large online sample [Preprint]. PLoS ONE.

[49] Killgore W.D.S., Cloonan S.A., Taylor E.C., Dailey N.S. (2020). Loneliness: A signature mental health concern in the era of COVID-19. Psychiatry Res..

[50] Luchetti M., Lee J.H., Aschwanden D., Sesker A., Strickhouser J.E., Terracciano A., Sutin A.R. (2020). The trajectory of loneliness in response to COVID-19. Am. Psychol..

[51] Walsh C. Young Adults Hardest Hit by Loneliness during Pandemic. https://news.harvard.edu/gazette/story/2021/02/young-adults-teens-loneliness-mental-health-coronavirus-covid-pandemic/.

[52] Kovacs B., Caplan N., Grob S., King M. (2021). Social networks and loneliness during the COVID-19 pandemic. Socius.

[53] Hawkley L.C., Hughes M.E., Waite L.J., Masi C.M., Thisted R.A., Cacioppo J.T. (2008). From social structure factors to perceptions of relationship quality and loneliness: The Chicago health, aging, and social relations study. J. Gerontol. Soc. Sci..

[54] Savikko N., Routasalo P., Tilvis R.S., Strandberg T.E., Pitkälä K.H. (2005). Predictors and subjective causes of loneliness in an aged population. Arch. Gerontol. Geriatr..

[55] Tilvis R., Jolkkonen K.P.J., Strandberg T. (2000). Social networks and dementia. Lancet.

[56] Elias S.M.S. (2018). Prevalence of loneliness, anxiety, and depression among older people living in long-term care: A review. Int. J. Care Sch..

[57] Drageset J., Kirkevold M., Espehaug B. (2011). Loneliness and social support among nursing home residents without cognitive impairment: A questionnaire survey. Int. J. Nurs. Stud..

[58] Singh A., Misra N. (2009). Loneliness, depression and sociability in old age. Ind. Psychiatry J..

[59] Kato T.A., Shinfuku N., Sartorius N., Kanba S. (2011). Are Japan’s Hikikomori and depression in younger people spreading abroad?. Lancet.

[60] Watts J. (2002). Public health experts concerned about “Hikikomori”. Lancet.

[61] Bäuerle A., Teufel M., Musche V., Weismüller B., Kohler H., Hetkamp M., Dörrie N., Schweda A., Skoda E.M. (2020). Increased generalized anxiety, depression and distress during the COVID-19 pandemic: A cross-sectional study in Germany. J. Public Health.

[62] Wu T., Jia X., Shi H., Niu J., Yin X., Xie J., Wang X. (2021). Prevalence of mental health problems during the COVID-19 pandemic: A systematic review and meta-analysis. J. Affect. Disord..

[63] Kikuchi H., Machida M., Nakamura I., Saito R., Odagiri Y., Kojima T., Watanabe H., Fukui K., Inoue S. (2020). Changes in psychological distress during the COVID-19 pandemic in Japan: A longitudinal study. J. Epidemiol..

[64] Noguchi T., Saito M., Aida J., Cable N., Tsuji T., Koyama S., Ikeda T., Osaka K., Kondo K. (2021). Association between social isolation and depression onset among older adults: A cross-national longitudinal study in England and japan. BMJ Open.

[65] Seginer R. (2000). Defensive pessimism and optimism correlates of adolescent future orientation: A domain-specific analysis. J. Adolesc. Res..

[66] Seginer R., Lilach E. (2004). How adolescents construct their future: The effect of loneliness on future orientation. J. Adolesc..

[67] Office for National Statistics (2018). What Characteristics and Circumstances Are Associated with Feeling Lonely.

[68] Nicolaisen M., Thorsen K. (2014). Loneliness among men and women: A five-year follow up study. Aging Ment. Health.

[69] Tijhuis M.A., De Jong-Gierveld J., Feskens E.J., Kromhout D. (1999). Changes in and factors related to loneliness in older men. The Zutphen elderly study. Age Ageing.

[70] Okun M.A., Keith V.M. (1998). Effects of positive and negative social exchanges with various sources on depressive symptoms in younger and older adults. J. Gerontol. Ser. B.

[71] Berg S., Mellström D., Persson G., Svanborg A. (1981). Loneliness in the Swedish aged. J. Gerontol..

[72] Algren M.H., Ekholm O., Nielsen L., Ersbøll A.K., Bak C.K., Andersen P.T. (2020). Social isolation, loneliness, socio-economic status, and health-risk behaviour in Deprived neighbourhoods in Denmark: A cross-sectional study. SSM Popul. Health.

[73] Kearns A., Whitley E., Tannahill C., Ellaway A. (2015). Loneliness, social relations and health and wellbeing in deprived communities. Psychol. Health Med..

